# Impact of claudin‐10 deficiency on amelogenesis: Lesson from a HELIX tooth

**DOI:** 10.1111/nyas.14865

**Published:** 2022-07-28

**Authors:** Nicolas Obtel, Adeline Le Cabec, Thè Nghia Nguyen, Eloise Giabicani, Stijn J. M. Van Malderen, Jan Garrevoet, Aline Percot, Céline Paris, Christopher Dean, Smail Hadj‐Rabia, Pascal Houillier, Tilman Breiderhoff, Claire Bardet, Thibaud Coradin, Fernando Ramirez Rozzi, Catherine Chaussain

**Affiliations:** ^1^ Université Paris Cité, URP2496 Pathologies, Imagerie et Biothérapies Orofaciales et Plateforme Imagerie du Vivant (PIV), FHU‐DDS‐net, IHMOA, Dental School Montrouge France; ^2^ AP‐HP Services de médecine bucco‐dentaire, Hôpitaux Universitaires Bretonneau (CRMR phosphore et calcium, filière OSCAR et ERN Bond) and Charles Foix, FHU DDS‐net Ile de France France; ^3^ Univ. Bordeaux, CNRS, MCC, PACEA, UMR 5199 Pessac France; ^4^ Department of Human Evolution Max Planck Institute for Evolutionary Anthropology Leipzig Germany; ^5^ Deutsches Elektronen‐Synchrotron DESY Hamburg Germany; ^6^ Sorbonne Université, CNRS, De la Molécule aux Nano‐Objets: Réactivité, Interactions et Spectroscopies (MONARIS) Paris France; ^7^ Department of Earth Sciences, Centre for Human Evolution Research Natural History Museum London UK; ^8^ Department of Cell and Developmental Biology University College London London UK; ^9^ Université Paris Cité, INSERM1163 Institut Imagine; APHP, Hôpital Necker‐Enfants Malades, Department of Dermatology, Reference Center for Rare Skin Diseases Paris France; ^10^ Université Paris Cité, Sorbonne Université, Centre de Recherche des Cordeliers, INSERM, CNRS‐ERL8228 Paris France; ^11^ APHP, Service de Physiologie, Centre de Référence des Maladies Rénales Héréditaires de l'Enfant et de l'Adulte (MARHEA), Hôpital Européen Georges Pompidou Paris France; ^12^ Charité Universitaetsmedizin Berlin, Division of Gastroenterology, Nephrology and Metabolic Diseases, Department of Pediatrics Berlin Germany; ^13^ Sorbonne Université, CNRS, Laboratoire de Chimie de la Matière Condensée de Paris Paris France; ^14^ Eco‐anthropologie (EA), Muséum national d'Histoire naturelle, CNRS Université de Paris Paris France

**Keywords:** apatite, claudins, enamel, renal dysfunction, tight junctions, xerostomia

## Abstract

In epithelia, claudin proteins are important components of the tight junctions as they determine the permeability and specificity to ions of the paracellular pathway. Mutations in *cldn10* cause the rare autosomal recessive HELIX syndrome (Hypohidrosis, Electrolyte imbalance, Lacrimal gland dysfunction, Ichthyosis, and Xerostomia), in which patients display severe enamel wear. Here, we assess whether this enamel wear is caused by an innate fragility directly related to claudin‐10 deficiency in addition to xerostomia. A third molar collected from a female HELIX patient was analyzed by a combination of microanatomical and physicochemical approaches (i.e., electron microscopy, elemental mapping, Raman microspectroscopy, and synchrotron‐based X‐ray fluorescence). The enamel morphology, formation time, organization, and microstructure appeared to be within the natural variability. However, we identified accentuated strontium variations within the HELIX enamel, with alternating enrichments and depletions following the direction of the periodical striae of Retzius. These markings were also present in dentin. These data suggest that the enamel wear associated with HELIX may not be related to a disruption of enamel microstructure but rather to xerostomia. However, the occurrence of events of strontium variations within dental tissues might indicate repeated episodes of worsening of the renal dysfunction that may require further investigations.

## Graphical abstract

Our study investigates a third molar collected from a HELIX (Hypohidrosis, Electrolyte imbalance, Lacrimal gland dysfunction, Ichthyosis and Xerostomia) female patient, by microanatomical and physicochemical approaches. The enamel morphology, formation time, organization, microstructure and overall elemental composition appear to be within the natural variability. However, accentuated strontium variations are found within the enamel and dentin, with alternating enrichments and depletions following the direction of the periodical striae of Retzius. This suggests that the *CLDN10* mutation, responsible for the HELIX syndrome, does not alter enamel formation. The strontium marked events may reflect the renal dysfunction and the resulting ionic imbalance.

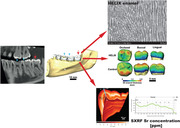

## INTRODUCTION

In mammals, tooth enamel is the most mineralized structure of the organism and forms the outer layer of the dental crown. Amelogenesis is a complex process that occurs before tooth eruption. It results from a complex epithelial–mesenchymal cross‐talk between the ectoderm‐derived enamel organ and the neural crest‐derived dental mesenchyme.^1^, ^2^, ^3^, ^4^ Enamel synthesis encompasses two major steps, namely, the secretory stage during which the ameloblasts, the enamel‐secreting cells of the enamel organ, secrete a template of enamel‐specific extracellular matrix proteins, and the maturation stage during which most of this scaffold is replaced by hydroxyapatite.^5^, ^6^, ^7^, ^8^, ^9^ Two types of maturation ameloblasts are reported according to morphological criteria.^9^, ^10^ Ruffle‐ended ameloblasts exhibit a distal plasma membrane with multiple invaginations, whereas smooth‐ended ameloblasts display a smooth distal membrane. These two cell types alternate during the maturation process and the pH of the associated enamel matrix from 6.2 for ruffle‐ended ameloblasts to 7.2 for smooth‐ended ameloblasts.^9^, ^11^, ^12^

At the secretory stage, ameloblasts display a double set of tight junctions (TJs), both at their apical and basal ends. At the maturation stage, smooth‐ended ameloblasts remove their apical TJs, whereas they reform in ruffle‐ended ameloblasts.^9^, ^10^, ^13^ Claudin proteins are the main components of the TJs that are either sealing the paracellular space or forming a pore, thus determining their permeability and ion specificity.^14^ The expression of several claudins has been reported in the secretory ameloblast TJs,^15^ including claudin‐1, ‐3, ‐16, and ‐19,^10^, ^16^, ^17^, ^18^ whereas claudin‐16 was not found in maturation ameloblasts.^16^ Claudin‐10 was shown to be expressed in the enamel organ and more precisely in the stratum intermedium, a layer of epithelial cells located immediately adjacent to the basal end of the ameloblast layer.^19^ Two isoforms of claudin‐10 are expressed in the kidney,^20^ claudin‐10a and claudin‐10b. The expression of claudin‐10a, which is anion‐selective, is restricted to the proximal tubule.^21^ Claudin‐10b, which is cation‐selective and may determine paracellular sodium permeability,^22^, ^23^ is expressed not only in the thick ascending limb of Henle's loop in the kidney^21^ but also in other epithelia.^24^, ^25^

Several genetic disorders affect the enamel structure of all the teeth from both dentitions, resulting in *Amelogenesis imperfecta* manifested by severe dental defects, which require complex restorations and significantly alter a patient's quality of life.^4^, ^5^, ^26^, ^27^, ^28^, ^29^ Among them, nonsyndromic *Amelogenesis imperfecta* are due to pathogenic variants of genes that encode enamel‐specific extracellular matrix proteins (*AMELX, ENAM*, and *AMBN)*, or proteins involved in enamel maturation (*MMP20, KLK4*, and *SLC24A4*), or cell–cell and cell–matrix attachments (*ITGB6, COL17A1, LAMA3*, and *LAMB3*).^26^, ^30^

*Amelogenesis imperfecta* is also frequently found in patients with genetic disorders related to kidney, skin, and other organs.^16^, ^26^, ^30^, ^31^ A disorder of this sort was recently found to be associated with loss‐of‐function variants of *CLDN10*, resulting in the autosomal recessive HELIX syndrome characterized by hypohidrosis, electrolyte imbalance, lacrimal gland dysfunction, ichthyosis, and xerostomia (OMIM 617671; prevalence: <1/1,000,000).^32^, ^33^, ^34^, ^35^, ^36^, ^37^ In addition, it has been reported that the patients with HELIX syndrome displayed a very early and severe enamel wear.^34^ At the time of examination, it was difficult to determine whether this severe enamel wear mainly resulted from erosion due to the impaired salivary secretion,^32^ or from enamel fragility directly related to claudin‐10 deficiency, as claudin‐10 is expressed in the forming tooth germ.^15^, ^19^ In the present study, the examination of the enamel of a retained third permanent molar, which was in a submucosal position and, therefore, partially exposed to the oral environment, was used to explore this question. The tooth was removed for orthodontic therapeutic reasons from a young female adult patient with HELIX syndrome. By combining microanatomical and physicochemical approaches, we showed that neither the rate of enamel formation nor its morphology, organization, and structure were significantly impacted by claudin‐10 deficiency. However, we identified the occurrence of random events of strontium variations within both enamel and dentin that may reflect a disorder in strontium handling, potentially caused by the renal dysfunction.

## MATERIALS AND METHODS

### Samples

An impacted right permanent third lower molar was collected from a 19‐year‐old female patient with the HELIX syndrome. This patient was already reported as patient A‐IV‐2.^34^ This family presented a missense variation c.386C >T (NM_182848), p.S129L in claudin‐10a (c.392C> T (NM_006984), and p.S131L in claudin‐10b. A 3D cone beam computed tomography (CBCT) exam of the lower jaw was performed to prepare the surgery. Since this impacted third molar displayed severely curved roots, the surgeon decided to section it to limit postoperative adverse events. Three impacted age‐matched third molars were gathered and were randomly used as control for the various experiments. All teeth were extracted at the request of an orthodontist in the context of a treatment plan and were collected with the informed consent of the patients, in accordance with the ethical guidelines laid down by French law (agreement IRB 00006477 and n° DC‐2009‐927, Cellule Bioéthique DGRI/A5). All teeth were fixed in 70% ethanol for a week.

### Preparation of the tooth sections

Sections of the crown of the HELIX patient's molar and control third molars were carried out to study the microanatomy of dental tissues by optical and scanning electron microscopy (SEM) and to perform chemical analyses by Raman microspectroscopy, energy‐dispersive‐X‐ray (EDX) microanalysis and synchrotron X‐ray fluorescence (SXRF) imaging. For the HELIX molar, since the distal half of the crown came as a fragment and was, therefore, more difficult to handle although it preserved both enamel and dentin, the section was performed on that fragment, thus yielding a bucco‐distal crown thin section. A bucco‐lingual section through the mesial cusps was performed on the control lower third molars.

For preparation of the thin sections for microanatomy, Raman, and SXRF analyses, the teeth were embedded in cyanoacrylate and fixed with wax on the glass slide. We used a saw equipped with a diamond disk (Struers, Champigny‐sur‐Marne, France) under a continuous water spray. After the first cut, the surface of the block was polished with carbide grinding paper (Grit 600/P1200) and Chemomet paper with 1 µm aluminum powder (Bühler, Uzwil, Switzerland). This surface was glued onto the slide with Araldite 2020 (Huntsman Corporation, The Woodlands, TX, USA). The block was then sectioned into ∼300 µm slices and polished (Grit 600/P1200) to reach an average thickness of ∼160 µm for the HELIX molar and ∼60 µm for the control third molar. The polishing process was kept minimal for the HELIX tooth because of its smaller size. Finally, the sections were polished again with Chemomet paper with 1 µm aluminum powder until a completely flat surface was obtained.

For preparation of the sections for SEM and EDX analyses, 1 mm‐thick sections in mirror of the control and HELIX third molars were prepared with a saw equipped with a diamond disk (Struers) under a continuous water spray. For SEM analysis, after thorough polishing, surfaces were cleaned with 5% sodium hypochlorite under ultrasonic activation for 2 min, rinsed twice with distilled water, etched with 36% orthophosphoric acid (DeTrey® Conditioner 36, Dentsply Sirona, York, PA, USA) for 12 s, and then thoroughly rinsed with distilled water. For EDX analysis, after polishing, surfaces were cleaned under ultrasonic activation for 2 min and rinsed twice with distilled water.

### Study of the microanatomy of dental tissues

The HELIX crown section was mounted on a glass slide for observation and analysis. The section was analyzed using incident light with a stereomicroscope Leica M8 and transmitted light with a Zeiss Universal photomicroscope. The Zeiss microscope was fitted with an Idea camera connected to a computer using Spot software (Version 5.4). The images were processed with Nikon ViewN2 and their analysis was performed with ImageJ.

Analysis of the dental microanatomy allows the study of the daily secretion rate (DSR) of the enamel and the formation time of the crown thanks to the presence of periodical growth lines in the enamel, the cross‐striations, and the striae of Retzius (Figure S1). Cross‐striations reflect the circadian variation of the enamel secretion, their spacing is indicative of the amount of enamel formed per day and yields the DSR.^38^ The striae of Retzius, which correspond to longer successive steps of enamel formation, are formed at regular intervals. Their periodicity is determined by counting the number of cross‐striations in between two successive striae. This periodicity is assumed to be constant during the entire crown formation time of a given tooth. According to the arrangement of Retzius' striae in the enamel, the dental crown can be divided into a cuspal part, located at the occlusal third of the tooth, in which striae are arranged in successive arches around the dentin horn and a lateral part, which is formed subsequently and until crown completion at the cervix^39^ (Figure S1). In the lateral enamel, striae of Retzius crop out and terminate at the surface of the enamel rather than arch over the dentin horn.

The DSR was obtained in the cuspal portion of the crown near the apparent dentin horn (Figure S1). A line running along the direction of an enamel prism between the enamel–dentin junction (EDJ) and the outer enamel surface (OES) was then divided into 100 µm‐thick zones to calculate DSR changes during the course of crown formation.^40^, ^41^ In each zone, the average spacing between cross‐striations was measured. This was performed several times in each zone, always across a minimum number of three cross‐striations, in order to obtain an average DSR for each zone, and finally to calculate an overall average DSR for the cuspal enamel. The total cuspal enamel formation time is equal to the thickness of cuspal enamel divided by the average daily cross‐striations spacing.

**FIGURE 1:**
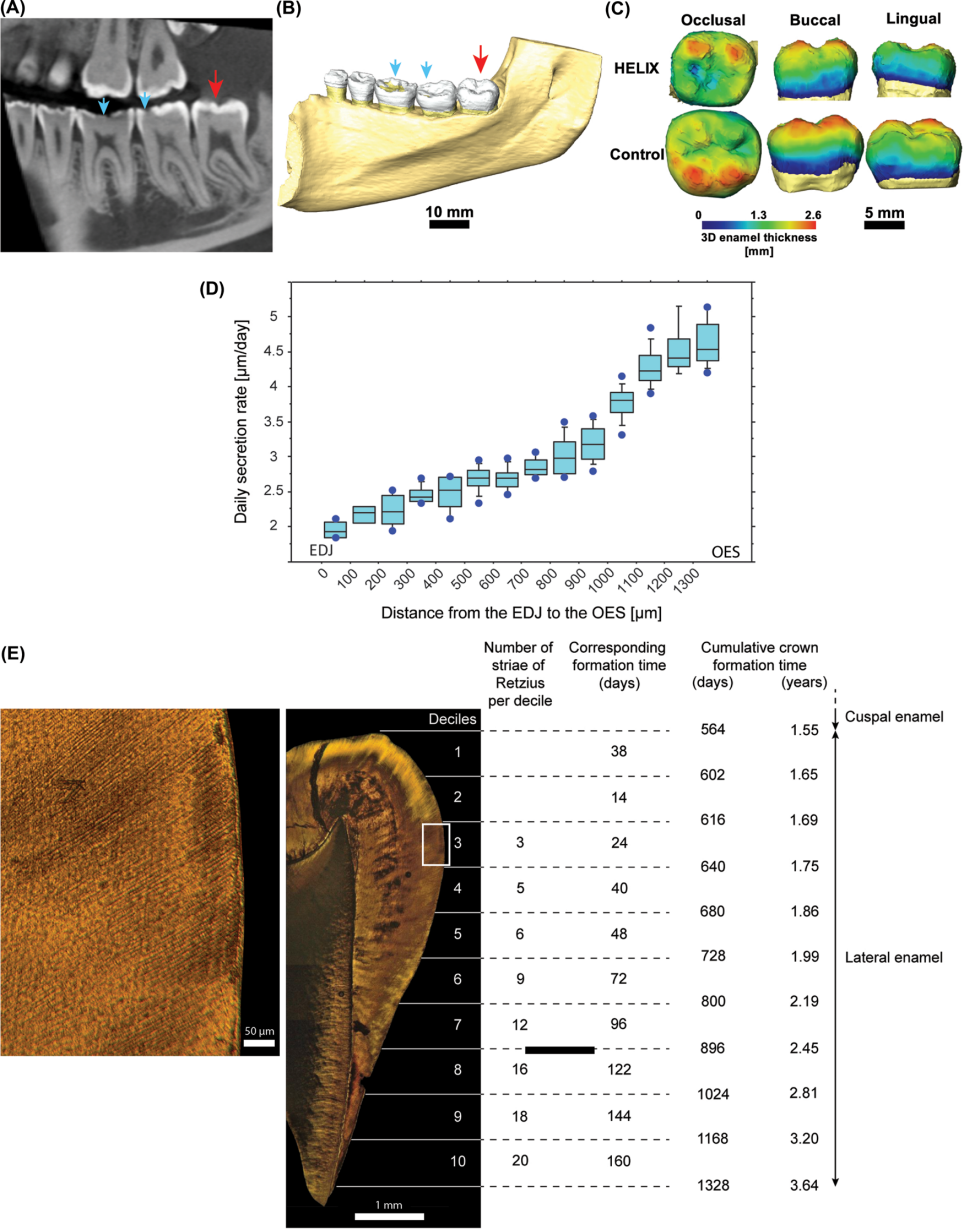
Anatomy and microanatomy of the HELIX molar. (A,B) Cross‐section of CBCT image (A) and 3D reconstruction (B) of the right lower jaw of the female HELIX patient. The partially erupted third molar (red arrow) shows an unworn and normally formed enamel, whereas the erupted molars display occlusal wear (blue arrows). (C) 3D reconstructions of the third molar crowns, showing that the HELIX enamel has a comparable 3D enamel thickness distribution to the control. (D) Determination of the daily secretion rate (DSR) in the HELIX enamel. The DSR (µm/day) increases from the enamel–dentin junction (EDJ) to the outer enamel surface (OES). (E) Quantification of the HELIX crown formation time. The cuspal enamel took 564 days (1.55 years) to form. The number of striae of Retzius and corresponding days of formation (days) are given for each decile of the lateral enamel. The total crown formation time is 1328 days (3.64 years). The area of interest symbolized by the white rectangle in the middle image is at higher magnification in the left image. See Figure S1 for details about the methodology.

To describe the development of the lateral enamel, the height of the crown, taken between the cusp tip and the enamel cervix, was divided into deciles of crown height.^42^ Noticeably, in the first two deciles, striae were difficult to distinguish so that the formation time was estimated by dividing the length of the prism path between the limit cuspal‐lateral enamel and the first striae of the third decile by the DSR of this area. The number of striae of Retzius was counted within each decile and multiplied by their periodicity. Periodicity was obtained in three locations. The lateral enamel formation time is equal to the total count of Retzius lines multiplied by their periodicity, that is, 8 days (Figure 1E). The crown formation time was obtained by the sum of cuspal and lateral enamel formation times (Figure S1).

### SEM imaging and EDX analyses

For SEM imaging, thick sections of the HELIX and control third molars were coated with a thin gold layer (*∼*30 nm) using a CRESSINGTON 108 AUTO gold sputter coater. For EDX measurements, the thick sections were coated with a thin layer of carbon (∼ 20 nm) by evaporation using a CRESSINGTON 208C carbon coater. Imaging and analysis were performed at 15 kV at different magnifications using a Hitachi SU‐70 microscope equipped with a Field Emission Gun. All the samples were evaluated for Ca, P, and Mg content (% atom) in the outer and inner layers of enamel, and at least three measurements were performed for each layer.

### Raman microspectroscopy

Raman analyses were performed using a Senterra Raman microspectrometer (Bruker Optics), using a laser emitting at 785 nm settled to provide a laser power at the sample of about 25 mW. Data collection was controlled by the OPUS 7.5 software (Bruker Optics). Measurements were collected across one spectral window (450–1800 cm^−1^) at a spectral resolution of about 3 cm^−1^, and each analysis was the coaddition of two spectra accumulated at up to 30 s of exposure time for each. Analyses were performed using a 50× objective (Olympus, Tokyo, Japan), giving an analytical spot size of approximately 12 µm in diameter, with the used excitation wavelength. Analyses were directly performed on polished surfaces of the thin sections. For mapping, the sample was moved by a computer‐controlled stage. Each surface was scanned by moving the sample by about 18 µm steps. Maps were performed on a 700 × 2150 µm surface for the control sample and 1000 × 1850 µm surface for the HELIX sample. Chemical maps were generated by integrating the area of the band centered at 1070 cm^−1^ (I_1070_) and the band centered at 960 cm^−1^ (I_960_), attributed to the vibration of carbonate (ν(CO_3_^2−^)) and phosphate (ν(PO_4_^3−^)) groups in the mineral phase, respectively.^43^ Ratios between both maps were done to monitor the chemical distribution. Maps have been depicted using the same color scheme. Full width at half maxima (FWHM) with imposed fitted position was also determined. Maps based on the ν(PO_4_^3−^) full width at half maxima (FWHM_960_) for both samples were also presented with a color scale going from 10 to 16 cm^−1^. Baseline subtractions, fitting, and map generation were managed by the OPUS 8.7 software (Bruker Optics).

### Synchrotron X‐ray fluorescence data acquisition and processing

SXRF analysis was performed on the P06 Beamline,^44^, ^45^ Petra III, at DESY (Deutsches Elektronen‐Synchrotron, Hamburg, Germany), a member of the Helmholtz Association HGF. Both the HELIX third molar and the control third molar were scanned. The thin section of the HELIX molar was left mounted on a glass slide support, while the control molar was mounted on suspended kapton foil. The storage ring was operated in 480‐bunch mode in top‐up filling mode with an average current of 120 mA ± 0.5 mA. The primary X‐ray beam was monochromatized to 16.6 keV using a double crystal Si111 monochromator and focused using a Kirkpatrick–Baez mirror system (JTEC, Japan) to approximately 500 × 500 nm^2^. The experimental configuration consisted of two Vortex EM silicon drift detectors (Hitachi High‐Tech Science America, Inc.), the second of which was collimated. Both detectors were positioned symmetrically at scattering angles of 135 degrees at a distance of 9 mm from the focal point at the sample surface. The use of dual‐detector “backscatter” geometry maximizes the solid angle during the analysis of thin polished samples (∼110 µm‐thick on average in this study), and allows large area to be scanned with micrometric resolution using millisecond dwell times.^46^ This setup allowed capturing Kα emission lines from Si to Sr, with varying detection efficiency.

Spectral peak deconvolution and integration were performed using the core of PyMca 5.5.0.^47^ Image analysis was performed in HDIP v‐1.3.3.1073 (Teledyne CETAC Technologies, Bozeman, MT, USA). The X‐ray yield calculations were performed using an in‐house script assuming a hydroxyapatite matrix (Ca_10_(PO_4_)_6_(OH)_2_) with density 2.85 g/cm^3^ for the enamel phase and 1.6 g/cm^3^ for the dentin phase.^48^ Elemental mass fractions were determined by calculating an areal density sensitivity from measurements standard Ti, Fe, and Cu foils with an areal density of 59.0, 55.0, and 47.9 µg/cm^2^, respectively (Micromatter Technologies Inc., Canada), and measured thickness of the samples. Tooth section thickness was measured throughout the whole surface of the specimens in four positions for HELIX and five positions for the control tooth. The average tooth section thickness (160 and 61 µm, respectively) was also taken into account in the X‐ray mass attenuation coefficients of the hydroxyapatite phase during attenuation correction.^49^ Glass slides and kapton foil substrates were included in the overall sample model as appropriate (i.e., background subtraction). Normalization to the incoming X‐ray flux was applied. In the calibrated data, SXRF concentrations are reported by mass fraction (µg.g^−1^, i.e., ppm), and/or areal density (g.cm^−3^).

A multiscale scanning strategy was used to optimize efficiency. First, a fast overview scan was acquired at 100 µm (dwell time: 10 ms) to check that the tooth section is well‐centered in the field of view, and assess the overall signal of the dental tissues. Then, a middle resolution (MR) overview scan allowed visualizing the elemental variation within the entire tooth section (i.e., enamel and dentin). The HELIX third molar section was scanned at 10 µm with 3 ms dwell time (X = 6.31 mm × Y = 6.44 mm, *t* = 33 min). The control third molar section was scanned at 10 µm, with 3 ms dwell time (X = 18.35 mm × Y = 7.99 mm, *t* = 1.3 h). Finally, based on prior observations of the HELIX tooth section under the microscope and the MR scans, two small regions of interest (ROI) were selected for acquiring high‐resolution (HR) scans: at 1.5 µm (dwell time = 4 ms): (1) across the lateral enamel just below the end of the cuspal enamel (ROI: X = 1.45 mm × Y = 0.37 mm, *t* = 18 min), and (2) across the EDJ roughly under the center of the occlusal basin (ROI: X = 1.21 mm × Y = 0.68 mm, *t* = 28 min). No HR scan was acquired on the control molar, due to a normal and monotonous signal in the MR scan. Visualization and analysis of the SXRF data were performed in HDIP. The color‐coded elemental maps were saved as 32‐bit tiffs allowing for a fine‐tuning of the contrast and brightness in ImageJ^50^, ^51^ to better reveal the stress pattern. To note that to denoise the images, a 2D Gauss filter was applied with a kernel size of 0.8 × 0.8.

### Variations in Sr content

Since the chronology of the crown formation was established, any variation in Sr concentration could be given a time relative to the initiation of crown formation. Each significant variation (i.e., enrichment or depletion) in Sr content was allocated a letter, their distance from the EDJ was measured, and their chronological order of formation calculated. The timing of the changes in Sr content between the EDJ and the enamel surface along a transect was quantified using the same methodology as used in the cuspal enamel, that is, the cumulative length of the prism between the two reference points was divided by the average DSR of the concerned area.

## RESULTS

### Physiopathological condition and phenotype of the HELIX patient

The HELIX patient is a 19‐year‐old French female (patient A‐IV‐2) born at full term from consanguineous parents, and was raised in France.^34^ She displayed xerosis of the skin with keratosis pilaris of cheeks, arms, thighs with a slight palmo‐plantar keratoderma, and xerostomia. As presented in Hadj‐Rabia *et al*.,^34^ she had normal serum calcium, high serum magnesium, low serum potassium concentrations, and hypocalciuria. The patient underwent an orthodontic treatment between 12 and 14 years of age and, at the time of dental examination, exhibited a metallic orthodontic retainer at the lower jaw from canine to canine. As shown by the clinical examination, the orthopantomogram^34^ and the CBCT examination (Figure 1A–C) of the right lower jaw, the patient exhibited severe enamel wear on all of her erupted teeth (blue arrows, Figure 1A,B). The crown of the third lower molar was fully formed although retained in a submucosal position and partially erupted into the oral cavity and displayed a normal morphology (red arrow, Figure 1A,B). In spite of the relatively low resolution of the CBCT scan (200 µm), the enamel thickness indices of the HELIX molar could be calculated on a virtual 2D section taken through the mesial cusps in the developmental plane (Figure 1C and Supplementary Information). These indices were within the range of the published values for modern human permanent third molars,^52^, ^53^ suggesting that the volume of formed enamel was not disturbed by claudin‐10 deficiency. Furthermore, CBCT showed that this third molar displayed an almost completed root formation and exhibited an enamel carious lesion located in the mesial fissure of the occlusal aspect (red arrow, Figure 1A).

### Daily secretion rate and crown formation time in the HELIX molar

The average DSR for each 100‐µm area in the HELIX molar is presented in Table S1. As shown in Figure 1D, the DSR increased from 1.96 µm/day in the inner zone of enamel near the EDJ to 4.62 µm/day in the outer zone of enamel near the enamel surface. The values and the pattern of the DSR for this HELIX third molar were similar to those reported for any normal human tooth.^54^, ^55^, ^56^

The total crown formation time of the HELIX tooth was then established (Figure 1E). For this tooth, we determined that the formation time of the cuspal enamel was 564 days. The periodicity of the striae of Retzius was 8 days. The number of striae in each decile of the lateral enamel is given in Figure 1E. The first two deciles corresponded to 52 days, while decile 3 to decile 10 contain 89 striae of Retzius formed over 712 days. The crown formation time was, therefore, 1328 days (3.64 years). The number of striae increased toward the cervix, from three striae in decile 3 (cusp tip) to 20 striae in decile 10 (cervix), indicating that the number of days within each decile increased toward the cervix (Figure 1E). In other words, the rate of crown lengthening slowed down from cusp tip to cervix. The pattern of striae of Retzius spacing among each of the deciles, as well as the formation time within each decile, and the overall crown formation time found in the HELIX third‐molar were similar to those already established for healthy modern human third molars.^42^

### Enamel microstructure of the HELIX tooth

**FIGURE 2:**
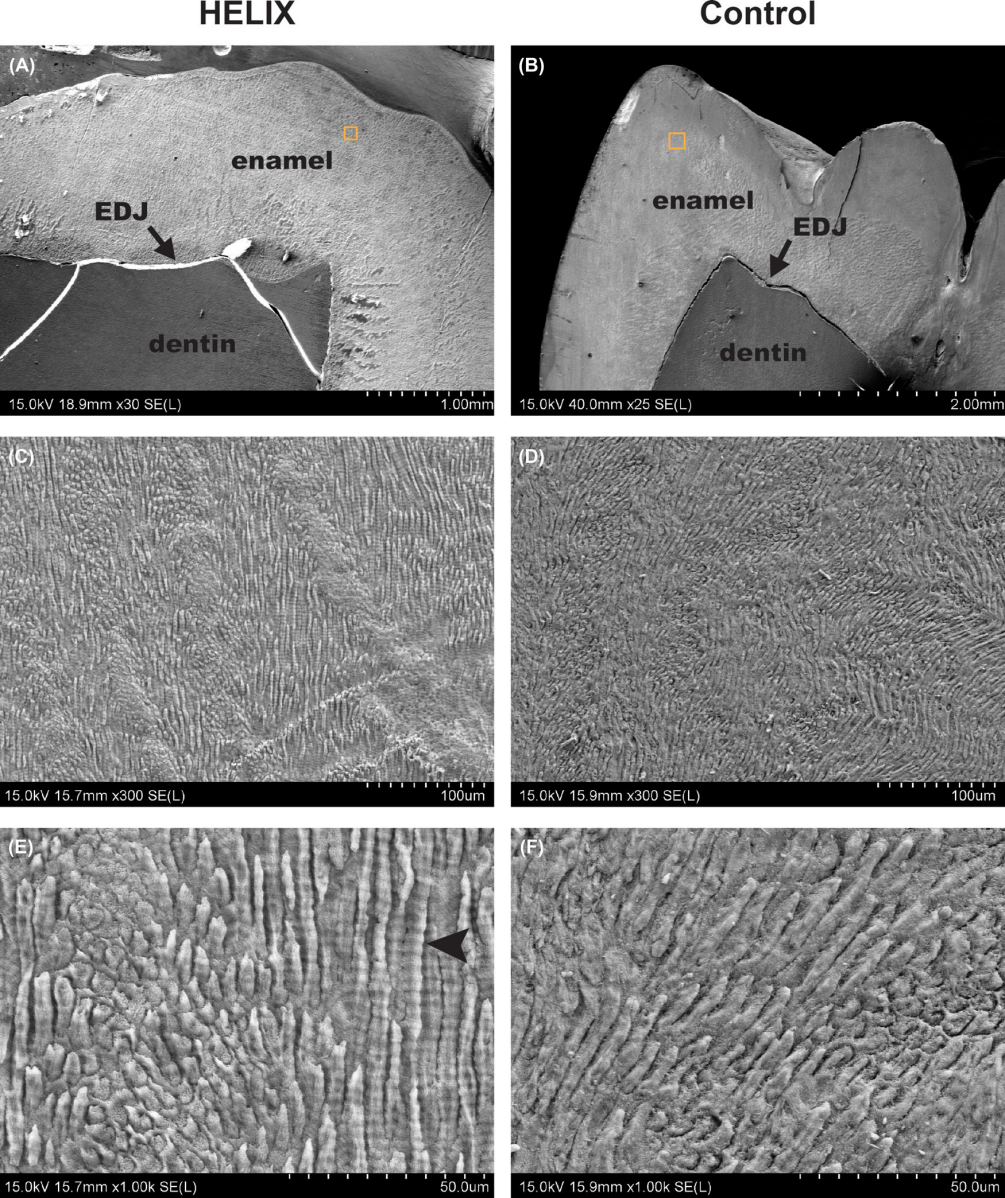
Characteristics of the HELIX enamel by SEM. (A–F) Representative aspects of the HELIX and control enamel microstructure imaged by SEM at various magnifications (A, B: ×25–30, C, D: ×300, E, F: ×1000, respectively). There is no difference between the HELIX enamel and the control enamel. Note that in the HELIX enamel, cross‐striations are particularly well‐distinguishable (E, black arrowhead). Abbreviation: EDJ, enamel–dentin junction.

We next investigated whether the microstructural characteristics of the enamel were affected by the HELIX syndrome. SEM observation showed that the enamel was correctly organized in HELIX when compared to control (Figure 2A,B). The enamel rods were perfectly formed and aligned in both cases (Figure 2C–F). Quite remarkably, the cross‐striations, which correspond to the circadian variation in enamel apposition,^38^ were particularly well‐distinguishable in the HELIX rods (Figure 2E, black arrow‐head), which may suggest a potential difference in the enamel content in HELIX. However, we cannot exclude that such variation may result from the sample processing, even if both teeth were prepared by the same operator, at the same time, and in the same conditions.^57^

### Crystallinity and carbonatation of the mineral phase

**FIGURE 3:**
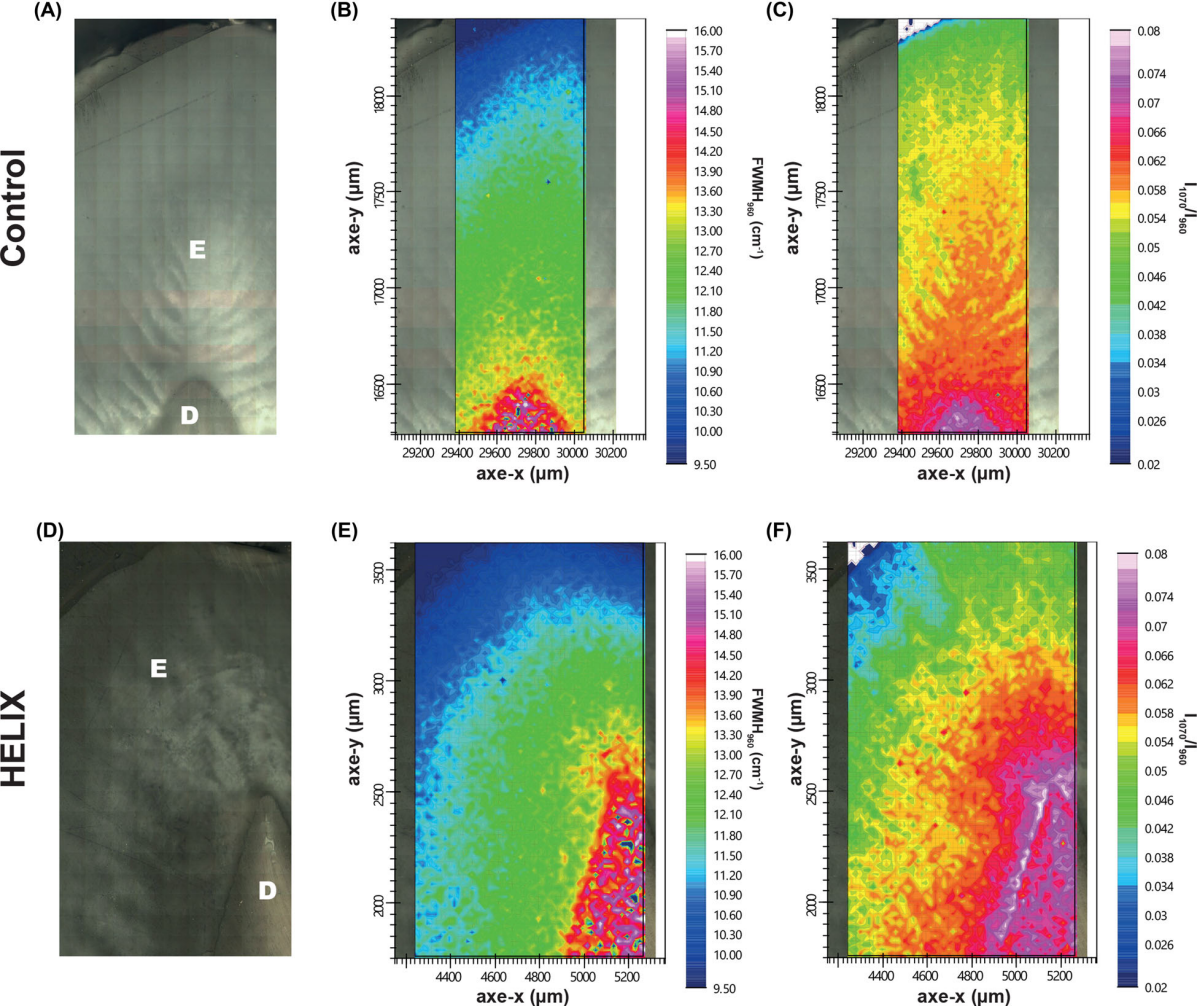
Characterization of the HELIX tooth using Raman microspectroscopy. (A,D) Optical microscopy of the imaged section of control and HELIX samples. (B,E) Raman mapping of the control and HELIX samples based on the full width at half maxima (FWHM) of the ν(PO_4_^3−^) vibration band at 960 cm^−1^. (C,F) Raman mapping of control and HELIX samples based on the ratio between the integrated area of the ν(CO_3_^2−^) and ν(PO_4_^3−^) vibration bands at 1070 and 960 cm^−1^, respectively. Abbreviations: D, dentin; E, enamel.

We then explored the mineral phase composition and structure of the HELIX dental tissues by Raman microscopy. Control and HELIX enamel samples were analyzed by recording Raman mappings based on the FWHM of the ν(PO_4_^3−^) band (Figure 3), indicative of the crystallinity of the apatite component of the tooth, and on the ratio between the integrated area of the ν(CO_3_^2−^) and ν(PO_4_^3−^) vibration bands, indicative of the carbonatation rate of this apatite component.^58^ The selected area extended from the outer enamel layer to the dentin core (Figure 3A,D). For the control sample, largest FHWM_960_ were found in the dentin area and, after an intermediate layer, the enamel part was characterized by a quite homogeneous, smaller FHWM_960_ value (Figure 3B). Similarly, the I_1070_/I_960_ ratio decreased from dentin to enamel but the area with intermediate values extended much significantly in the latter tissue (Figure 3C). These results are consistent with the fact that the mineral phase of enamel is a highly crystalline hydroxyapatite with low carbonatation rate, whereas the mineral phase of dentin has a higher degree of substitution and lower crystallinity.^59^ No significant difference in the evolution of both FHWM_960_ (Figure 3E) and I_1070_/I_960_ (Figure 3F) values from dentin to enamel could be evidenced in the HELIX sample compared to the control, suggesting the absence of modification of the crystallinity and carbonatation degree of the mineral phase in these two tissues.

### Chemical analyses

An investigation on the chemical composition of the enamel was performed to determine if it was altered, which would indicate an abnormal maturation and contribute to explaining the rapid enamel wear observed in all the patients with the HELIX syndrome.^34^ EDX analysis revealed that the Ca/P ratio was slightly lower in the HELIX enamel, especially in the outer layer, when compared to the control enamel (outer enamel layer: 1.54 vs. 1.58; inner layer: 1.59 vs. 1.60, respectively), and this was mainly due to a slightly lower Ca content (Table 1; Figures S2 and S3). As most of the patients with HELIX syndrome have been reported to have hypermagnesemia,^32^, ^34^ the (Ca + Mg)/P ratio was also calculated, revealing a similar pattern in both enamel samples (Table 1). No differences were found for the dentin by either SEM (Figure S4) or EDX (data not shown).

**TABLE 1:** Atomic quantification of elements (in At [%]) in HELIX and control in the inner and outer layers of enamel by EDX analyses

	HELIX			Control		
Element	P	Ca	Mg	P	Ca	Mg
**Outer**						
Max	13.7	21.0	0.2	13.7	21.6	0.2
Min	13.5	20.9	0.1	13.5	21.4	0.1
Average	13.6	21.0	0.2	13.6	21.5	0.2
Standard deviation	0.1	0.1	0.0	0.1	0.1	0.0
Ca/P	1.54			1.58		
(Ca+Mg)/P	1.55			1.59		
**Inner**						
Max	13.6	21.4	0.5	14.0	22.4	0.4
Min	12.7	20.2	0.4	13.8	22.1	0.4
Average	13.0	20.7	0.4	13.9	22.2	0.4
Standard deviation	0.5	0.7	0.1	0.1	0.2	0.0
Ca/P	1.59			1.60		
(Ca+Mg)/P	1.62			1.62		

**FIGURE 4:**
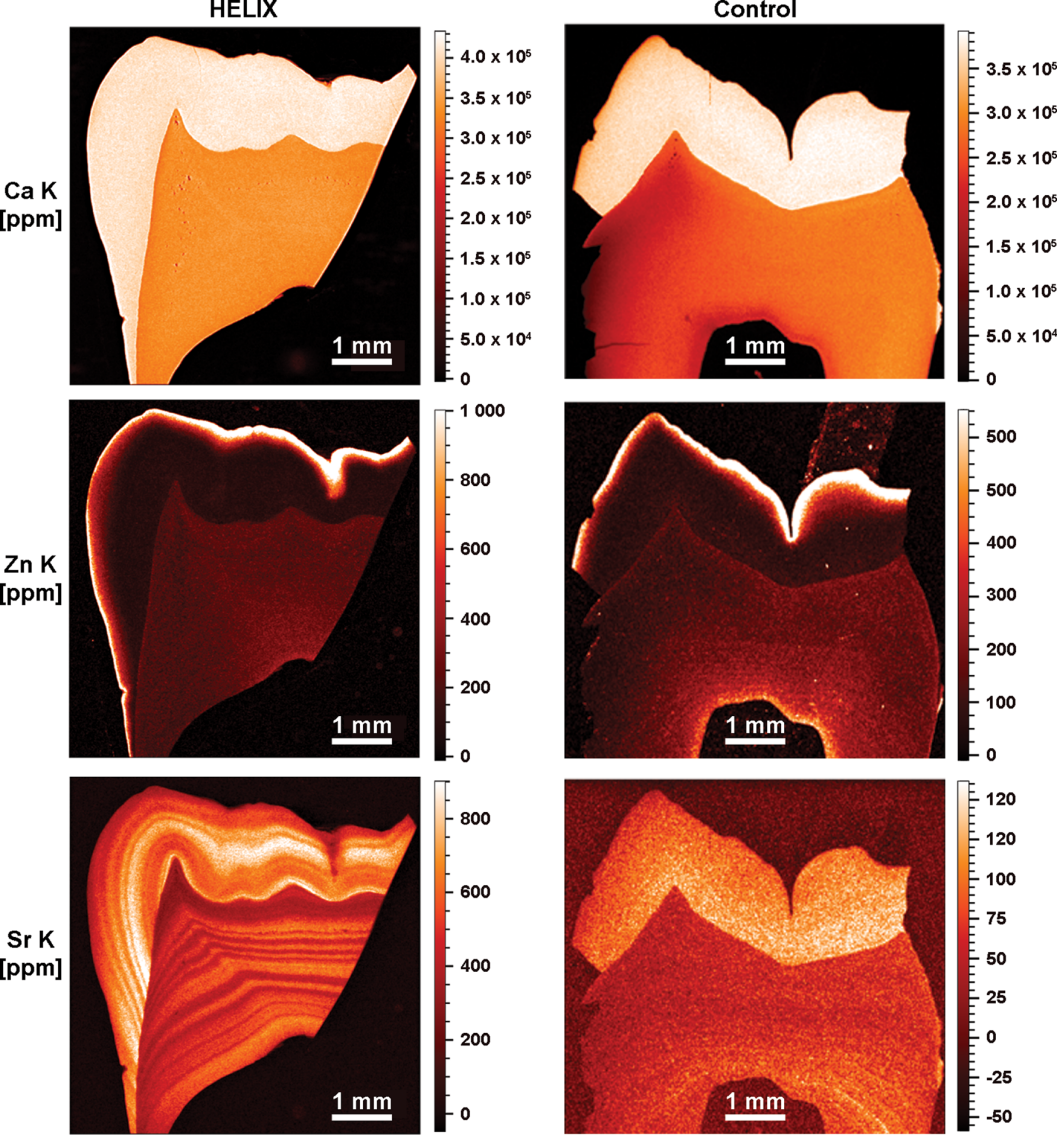
SXRF characterization of the HELIX molar. SXRF characterization of the HELIX (left) and control (right) teeth for mapping Ca (upper panel), Zn (middle panel), and Sr (lower panel) (SXRF overviews at 10 µm). In both the HELIX and control molar crowns, Ca and Zn have a similar distribution with comparable ranges of concentration (in ppm). HELIX shows Sr levels that are six times higher than in the control, with marked patterns of alternating Sr enrichments and depletions in both the enamel and the dentin.

Next, multielement analysis of the samples was performed using SXRF to investigate further possible modifications of the chemical composition of enamel induced by the HELIX syndrome (Figure 4).

#### Calcium

In the HELIX tooth, the SXRF levels of Ca in enamel and dentin are uniform and reach on average 4.0 × 10^5^ ppm and 3.4 × 10^5^ ppm, respectively (Figure 4, left upper panel). In the control tooth (permanent third molar), Ca levels range between 3.6 × 10^5^ and 3.85 × 10^5^ ppm in enamel, and between 2.0 × 10^5^ and 2.8 × 10^5^ ppm in dentin (Figure 4, right upper panel). These Ca values are compatible with the published values of enamel and dentin imaged by SXRF, ranging from 3.8 × 10^5^ to 5.0 × 10^5^ and from 3.1 × 10^5^ to 3.7 × 10^5^ ppm, respectively.^60^, ^61^, ^62^, ^63^ The present results show that *CLDN10* deficiency did not alter significantly the process of tooth tissue mineralization.

#### Zinc

Zn levels imaged by SXRF in the first‐formed inner enamel are relatively low. They rise in a steep gradient toward the OES, where Zn levels are greatly enriched. In HELIX tooth, the OES at the occlusal basin reaches 1.8 × 10^3^ ppm, while at the lateral enamel surface, it peaks at 1.2 × 10^3^ ppm (Figure 4, left middle panel). The middle and inner enamel is at ∼100 ppm, while the dentin is at ∼220 ppm. In the control sample, although the enamel cap is not fully preserved, Zn values at the OES peak at ∼1.0 × 10^3^ ppm on the occlusal aspect of the cusps, while it is slightly less on the surface of the lateral enamel (600–800 ppm). The inner and middle zones of enamel contain ∼60 to ∼100 ppm of Zn, while the dentin is at ∼200 ppm. Zinc enrichment at the OES has been previously described as a normal feature, potentially related to the processes of enamel mineralization and maturation.^60^, ^64^, ^65^ SXRF values in human deciduous teeth show peak values ranging from 400 to 500 ppm at the OES,^60^ while in *Pongo*, the outer layer of enamel concentrates 1.5 × 10^3^ to 2.0 × 10^3^ ppm of Zn.^66^ Rautray *et al*.^67^ reported Zn values on human healthy enamel yielding an average of 172.2 ppm, which is within the same order of magnitude as the present middle and inner values measured in HELIX and control.

#### Strontium

**FIGURE 5:**
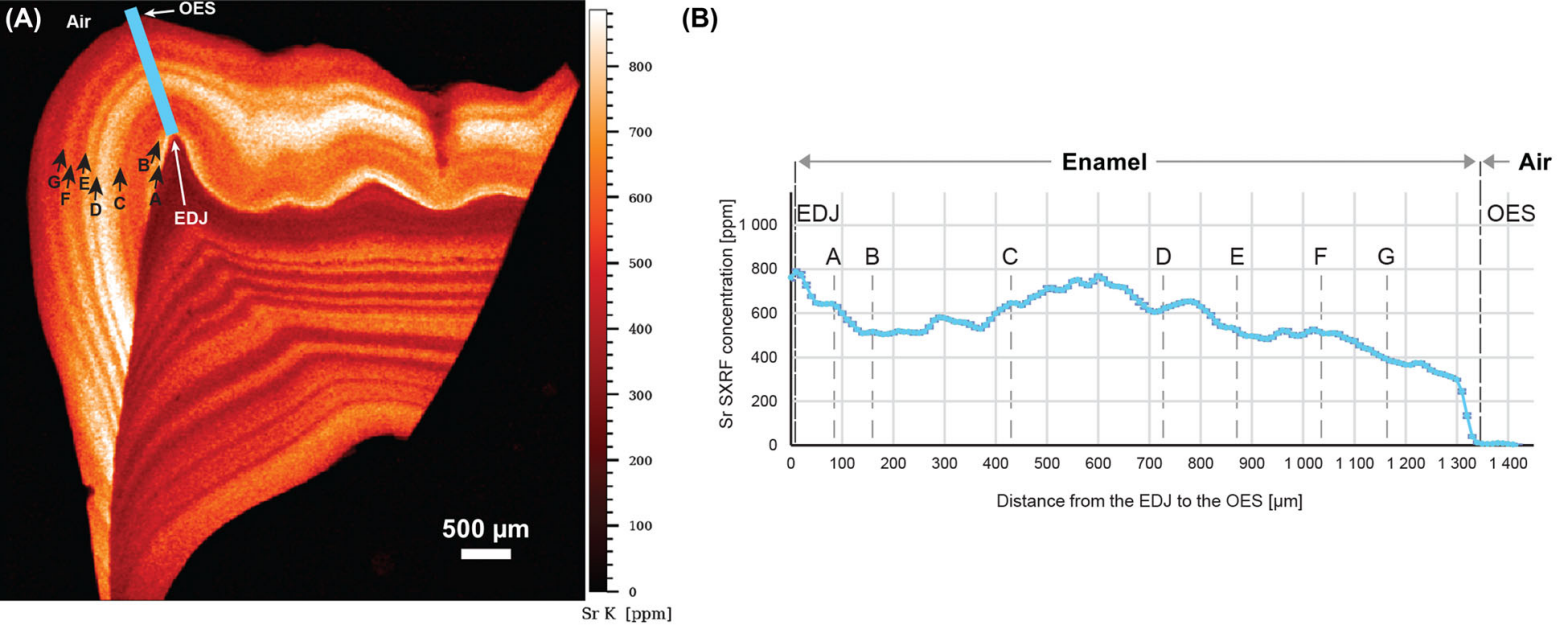
Variations in Sr content in the cuspal enamel of the HELIX tooth. (A) Sr SXRF map of the HELIX tooth showing the variation in Sr content in enamel and dentin. Datapoints were collected along the blue transect to plot Sr concentrations as a function of distance (see B) from the enamel–dentin junction (EDJ) to the outer enamel surface (OES). Marked Sr variations following the direction of the enamel growth lines were attributed letters (from A to H; black arrows). There is no clear temporal periodicity in the occurrence of these Sr variations.

In the control tooth, the Sr distribution (Figure 4, right lower panel) followed previously published observations.^60^ On average, Sr levels in enamel ranged from 50 to 130 ppm (outer vs. inner enamel, respectively), which is compatible with an average of 174.76 ppm calculated from values provided in Ref. 67. In dentin, Sr values in the control tooth ranged between 40 and 80 ppm, with an accentuated event at 150 ppm. In the HELIX sample (Figure 4, left lower panel), a strong pattern of variation in Sr concentrations was visible using SXRF in both the enamel and dentin, from the earliest stages of formation of the third molar until crown completion and beyond into the root dentin. An alternation of strong depletions and enrichments occurred with a high frequency. Both HR SXRF scans at 1.5 µm confirmed that these accentuated Sr markings occurred simultaneously in enamel and dentin (Figure S5). As the abrupt changes in the content of Sr followed the direction of the striae of Retzius (Figure 5A), further investigation was performed to determine whether these events take place at specific periods during the crown formation, with no overprinting from subsequent Sr ingestions.^66^ Within the ∼1330 µm of cuspal enamel, these several episodes of Sr variation were each calculated to last from 33 to 121 days, (Figure 5B and Table S2). Within enamel, these bands of Sr variations peaked at 860 ppm (direct measurement in HDIP v‐1.3.3.1073, outside of the blue transect in Figure 5A) in the middle of the cuspal enamel, while the strongest depletions dropped down to ∼300–350 ppm, especially at the OES (Figure 4, left lower panel). In dentin, values were lower with peaks at 600–650 ppm and troughed at 280–340 ppm (Figure 4, left lower panel). The variation in Sr concentration as well as their timing in days did not suggest that these changes followed any regular and periodical pattern. These findings prompted us to question the HELIX patient on a particular exposure to strontium at any time of her growth, including the period corresponding to the third molar formation. She did not report either a specific diet or using any specific toothpaste enriched in strontium ions to prevent tooth hypersensitivity.

#### Other elements

Other elements with Kα lines in the detectable energy range, which include Cu, Fe, Mn, Ti, Cr, S, Cl, Ar, and Rb, did not yield any significant detectable differences between the HELIX and the control tooth in terms of elemental distribution or abundance (Figure S6).

## DISCUSSION

The HELIX syndrome is a very rare disorder (OMIM 617671; Prevalence: <1/1,000,000), which manifests as abnormalities in renal ion homeostasis, resulting in hypermagnesemia, hypocalciuria, and hypokalemia, in epidermal integrity and homeostasis of the ectodermal glands, including salivary glands. We previously reported that all patients with HELIX syndrome displayed early and severe enamel wear,^34^ compromising their oral health and particularly their chewing capacity. The present study is based on the observation of a third molar from a patient with HELIX syndrome, which was collected prior to the full eruption of the tooth and, therefore, before the crown was fully exposed to the challenges of the environment of the oral cavity,^68^, ^69^, ^70^ and shows that the enamel was correctly formed and displayed normal maturation.

The general pattern of enamel formation appears to be consistent with that described for normal human permanent molars.^54^ Specifically, the DSR gradient increase from the EDJ to the surface in the cuspal enamel matches that in normal third permanent molars. The time taken to form lateral enamel is also within the ranges reported for human third permanent molars,^42^ and the total enamel formation time is within the ranges reported in the literature.^42^, ^52^, ^71^, ^72^ It seems unlikely, therefore, that the HELIX syndrome has any impact on the timing of enamel formation.

The structure of enamel in both HELIX and control samples was studied both at the microscale by SEM and Raman spectroscopy. Although these techniques were previously shown to clearly characterize other dental disorders, such as *Amelogenesis imperfecta* ^73^, ^74^ or X‐linked hypophosphatemia,^75^ no significant difference could be found in the present study between the HELIX and the control molars.

Similarly, the EDX determination of the Ca, P, and Mg content led to similar values for both samples. However, this technique has a rather high detection limit (*∼*1000 ppm), especially for light elements. In contrast, SXRF allows for quantification below 1 ppm and was already applied for multielement analysis of trace elements in many biological tissues,^76^ including teeth.^60^, ^66^ Here, among the many collected elements, Sr stood out as the only investigated element showing a clear difference in amount and distribution between the two samples. Not only its local concentration could be six times higher in the enamel of the HELIX tooth compared to the control, but it also formed well‐defined zones of enrichment and depletion parallel to the striae of Retzius, attesting that this chemical signal has been integrated to the dental tissues during development. However, in contrast to these periodical growth markings, no periodicity could be found in the occurrence of these Sr bands, suggesting that Sr incorporation is not linked to a specific, regular step of amelogenesis. In fact, it is notable that Sr bands are also present synchronously in the dentin, suggesting that Sr presence is related to an overall mineral homeostasis disorder rather than to a local disturbance of amelogenesis.

Dental hard tissues contain trace elements of both dietary and environmental origin.^77^ Among them, strontium ions are divalent cations and thus can easily substitute for Ca in the hydroxyapatite structure or interact with the mineral phase.^78^ It has been previously reported that the substitution of Ca by Sr in hydroxyapatite alters its solubility.^79^ However, this was demonstrated for Sr amount above 1%,^79^ which is not the case in the present tooth analysis, where the substitution appears to occur at 0.1%. Therefore, although we cannot fully exclude a modification of apatite solubility with such a low substitution rate, it is expected to be very minor. *In vivo*, it was found that by oral treatment of increasing Sr dose in rats, it was possible to obtain Sr/(Sr + Ca) molar ratio > 0.01 in femur without altering the bone structure.^80^ As a comparison, the Sr/(Sr + Ca) molar ratio here was *∼*0.002, which could explain the absence of a clear difference in the enamel structure between HELIX and control in this study. Accordingly, it was not possible to detect Sr‐related spatial variations of Ca amount in the Ca SXRF map. A possible explanation of this high and random adsorption of Sr within enamel and dentin might be an excess of strontium consumption in the patient's diet or by swallowing toothpaste.^81^ However, our HELIX patient, who was born and raised in France, did not report either a specific diet or using, at any time of her growth, including the period corresponding to the third molar formation, any specific toothpaste enriched in strontium ions to prevent tooth hypersensitivity.

The renal clearance of Sr can be computed to 2–3 ml/min in normal subjects. It is much lower than normal glomerular filtration rate, indicating that Sr excreted in urine is only a tiny fraction of filtered Sr: therefore, most of Sr filtered at the glomerulus is reabsorbed along the renal tubule, as Ca and Mg are.^82^, ^83^ Patients with HELIX syndrome have very low urinary calcium and magnesium excretion in urine, despite normal serum Ca and high serum Mg levels, indicative of increased tubular Ca and Mg reabsorption.^32^ That Sr is also excessively reabsorbed across the renal tubular epithelium is a sound hypothesis, albeit not documented. Subsequently, patients with HELIX syndrome may have a higher than normal serum Sr concentration, all the more so since serum Sr level increases when GFR decreases.^82^ Thus, a higher serum Sr level may result in a higher deposition of Sr in bone and tooth. Future studies are needed to investigate whether abnormal levels of Sr are also identified in bones of HELIX syndrome patients and, more broadly, how the kidney dysfunction associated with the HELIX syndrome impacts plasma and urine Sr concentrations.

One of the main functions of the enamel organ during the maturation stage is to transport very large amounts of mineral ions, especially calcium and phosphate, from the blood vessels to the enamel matrix. This critical process is finely controlled by a great number of ion channels, transporters, and exchangers.^10^, ^11^, ^84^, ^85^ In the HELIX syndrome, one of the consequences of the renal dysfunction is hypocalciuria.^32^

The precise role of Mg in amelogenesis remains not fully understood, yet the expression of the Mg transporter CNNM4 in ameloblast cell membranes at the transition and maturation stages supports that Mg might be removed from the enamel matrix to promote mineralization.^86^ It has indeed been consistently reported that Mg content of the enamel is inversely correlated with the extent of mineralization.^87^, ^88^ Here, the magnesium content was found to be normal in the HELIX enamel, suggesting that the hypermagnesemia found in the HELIX patients^32^ may not have direct consequence on enamel mineralization.

Hypokalemia is a frequent feature of the HELIX patients.^32^ Potassium ions have been shown to be important for normal enamel formation as several ion exchangers or cotransporters are K^+^‐dependent.^10^, ^11^, ^84^, ^89^ For example, the alteration of the K^+^‐dependent Na^+^/Ca^2+^ exchanger isoform 4 (*SLC24A4*) into mouse maturation ameloblasts by an excess of fluoride in the drinking water was shown to impair amelogenesis.^89^ Furthermore, patients with syndromes associated with hypokalemia, such as the Bartter's syndrome, were reported to display *Amelogenesis imperfecta*.^90^, ^91^ It is, therefore, likely that the hypokalemia measured in the patients with the HELIX syndrome may contribute to higher enamel fragility.

Taken together, our data show that enamel formation is not significantly impaired by *CLDN10* deficiency, rather designating xerostomia as the main culprit of the enamel wear found in HELIX patients. However, the abnormal concentrations of Sr measured in the dental mineralized tissues suggest that the tooth mineral content may reflect repeated episodes of worsening of renal dysfunction. It cannot be denied that such changes in Sr concentrations, albeit very low, may alter enamel and dentin solubility. These events urge the need to investigate more teeth from HELIX patients. Serum strontium concentration should also be monitored. Nevertheless, the study of a murine model of the HELIX syndrome may provide further insights on the direct role of claudin‐10 in amelogenesis.

## AUTHOR CONTRIBUTIONS

C.C., F.R.R., T.C., and T.B. conceived and designed the study. F.R.R., N.O., T.N.N., and E.G. prepared the tooth thin‐sections. J.G., A.L.C., M.C.D., and S.J.M.V.M. acquired the SXRF data. S.J.M.V.M. and J.G. postprocessed the SXRF data. A.P. and C.P. recorded the Raman data. A.P., C.P., and T.C. interpreted the Raman data. C.C., N.O., and T.C. interpreted the SEM and EDX data. A.L.C., F.R.R., and M.C.D. analyzed and interpreted the SXRF data. C.C., N.O., A.L.C., C.B., T.C., P.H., S.H.R., and F.R.R. drafted the manuscript, and with revisions and final approval of the submitted version by all coauthors.

## COMPETING INTERESTS

The authors declare no competing interests.

### PEER REVIEW

The peer review history for this article is available at: https://publons.com/publon/10.1111/nyas.14865.

## Supporting information

Supplementary Information: Quantification of enamel thickness.

**Figure S1** Schematic of the methodology for the quantification of the HELIX crown formation time

**Figure S2** Analysis of the HELIX enamel layers by EDX. EDX atomic mass spectra of the outer (B) and inner (C) enamel. Area of interest is shown (A) (yellow: outer, inner: green).

**Figure S3** Analysis of the control enamel layers by EDX. EDX atomic mass spectra of the outer (B) and inner (C) enamel layers. Area of interest is shown (A) (yellow: outer, inner: green).

**Figure S4** Characteristics of the HELIX dentin by SEM. Representative aspects of the HELIX (A) and control (B) dentin microstructure imaged by SEM at x300 magnification. There is no difference between the HELIX dentin and the control dentin.

**Figure S5** Location of the high‐resolution SXRF scans (1.5 µm of resolution) on the transmitted light image of the HELIX tooth section (top left), and Ca, Sr, and Zn maps of the high‐resolution scans acquired at the EDJ (A), and in the lateral enamel (B). The fine variations in Sr seen on the overview maps (Figure 4) are also detectable at higher power, both in enamel and dentin. E, enamel; A, air; PD, primary dentin; EDJ, enamel–dentin junction. In all SXRF maps, the scale bar is 100 µm long.

**Figure S6** SXRF characterization of the HELIX molar. SXRF characterization of the HELIX and control teeth for mapping Fe, Cu, Mn, Ti, Cr, Br, Ar, S, Rb, and Cl (SXRF overviews at 10 µm). In both the HELIX and control molar crowns, all these elements have a similar distribution with comparable ranges of concentration (in ppm).

**Table S1** Daily secretion rate (DSR) from the enamel–dentin junction (EDJ) to the outer enamel surface (OES)

**Table S2** Cumulative formation time and distance from the outer enamel surface (OES) to the enamel–dentin junction (EDJ) of the major strontium (Sr) variations evidenced by synchrotron X‐ray fluorescence (SXRF)
