# Supplementary material for: Impact of claudin‐10 deficiency on amelogenesis: Lesson from a HELIX tooth
Source: Ann N Y Acad Sci. 2022 Jul 28;1516(1):197–211. doi: 10.1111/nyas.14865 (PMC9796262; doi:10.1111/nyas.14865)
Supplement: Supplementary file 1 — Supplementary Information: Quantification of enamel thickness. [file NYAS-1516-197-s008.docx]

**Supplementary Information**

**Impact of Claudin 10 deficiency on amelogenesis: lesson from a HELIX tooth**

Nicolas Obtel^†^, Adeline Le Cabec^†^, Thè Nghia Nguyen, Eloise Giabbicani, Stijn J. M. Van Malderen, Jan Garrevoet, Aline Percot, Céline Paris, Christopher Dean, Smail Hadj Rabia, Pascal Houillier, Tilman Breiderhoff, Claire Bardet, Thibaud Coradin, Fernando Ramirez Rozzi, Catherine Chaussain.

**Quantification of enamel thickness**

A. *Enamel thickness indices in Dental Anthropology*

The enamel distribution in tooth crowns has been extensively investigated by dental anthropologists ^e.g., 1–13^, first for taxonomic purposes, then for discussing dietary adaptions, developmental patterns and pathologies.

Indices in 2D and 3D have been designed to capture the enamel distribution on the dentine core of the crown; they involve the 2D or 3D Average Enamel Thickness (AET) index (expressed in mm), and the Relative Enamel Thickness (RET) index (scale-free index)^1,14^.

Here we propose to quantify these indices in the HELIX mandibular right third molar (LRM3), based on the cone-beam CT scans acquired on the HELIX patient prior to tooth extraction. We are aware that the low resolution of the scan (200 µm) may affect the accuracy of the measurements, especially regarding the detection of the EDJ. Another source of tissue proportion underestimation might be the carious lesion affecting the occlusal basin in the mesial aspect of the crown (See Fig. 1A, B, C). This is however only intended to show that, compared to measurements performed on healthy modern human molars, the HELIX tooth shows a normal enamel thickness, with a normal distribution. To quantify whether HELIX fits within this healthy modern variation, we compute adjusted z-scores, *Azs* ^15,16^. This statistics enables to compare a specimen to a reference sample. If *Azs*=0, the HELIX tooth is close to the mean of the healthy modern humans. If *Azs*>1 or *Azs*<1, HELIX falls outside of the healthy modern human variation, and is greater or smaller than the comparative variability, respectively. If -1<*Azs*<0, HELIX falls within the lower end of the healthy modern human variation, while if 0<*Azs*<1, HELIX falls within the upper end of their variation.

*B. 2D Enamel Thickness*

*B.1. 2D enamel thickness indices in the HELIX M3s*

For the 2D indices (2D AET and 2D RET), a virtual 2D section passing through the developmental plane of the mesial cusps is recorded, and the surface areas of enamel and dentine are measured, as well as the EDJ length and the bicervical width. The 2D indices are calculated as follows:

2D AET = SA_Enamel_ / Length_EDJ_

2D RET = 2D AET / (SA_Dentine_^1/2) × 100


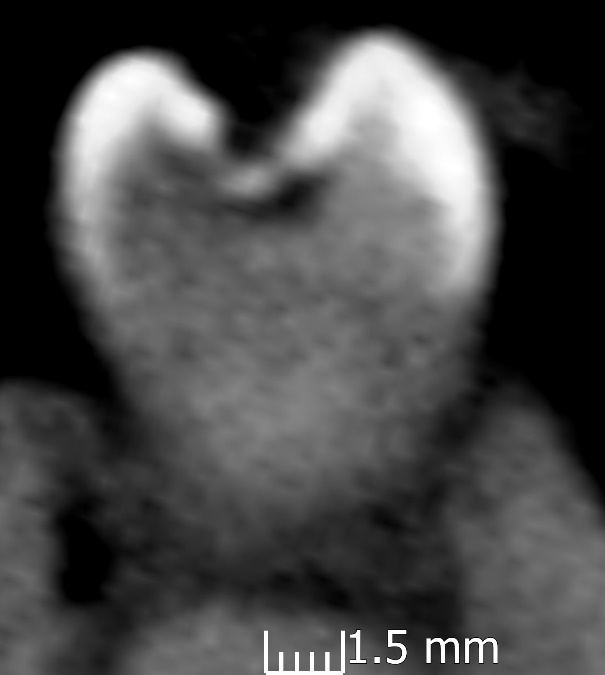


Virtual 2D section passing through the developmental plane of the mesial cusps of the HELIX LRM3.

For the sake of completeness, we also measured these indices on the left third molar of the HELIX patient.

| Specimen | SA_Cr_ [mm²] | SA_Dent_ [mm²] | SA_Enam_ [mm²] | L_EDJ_ [mm] | 2D AET [mm] | 2D RET [scale free] | BW [mm] |
| --- | --- | --- | --- | --- | --- | --- | --- |
| HELIX LLM3 | 43.11 | 24.30 | 18.81 | 16.97 | **1.11** | **22.48** | 7.07 |
| HELIX LRM3 | 39.40 | 19.40 | 20.00 | 15.28 | **1.31** | **29.72** | 6.68 |

SA_Cr_: whole Crown Surface Area; SA_Dent_: Dentine Surface Area; SA_Enam_: Enamel Surface Area; S_EDJ_: EDJ length; 2D AET: 2D Average Enamel Thickness; 2D RET: 2D Relative Enamel Thickness; BW: bicervical width.

*B.2. Comparison of the HELIX LM3s to healthy modern M3s (source: Smith et al., 2006) using adjusted z-scores ^15,16^.*

|  |  | SA_Dent_ [mm²] | SA_Enam_ [mm²] | L_EDJ_ [mm] | 2D AET [mm] | 2D RET [scale free] | BW [mm] |
| --- | --- | --- | --- | --- | --- | --- | --- |
| *Comparative sample of modern human M3*^17^ | | | | | | | |
|  | N | 44 | 44 | 44 | 44 | 44 | 44 |
|  | Mean | 33.09 | 22.58 | 18.27 | 1.24 | 21.63 | 8.53 |
|  | SD | 5.11 | 3.28 | 1.36 | 0.15 | 2.99 | 0.74 |
| *Adjusted z-scores* | | | | | | | |
| Azs HELIX LLM3 | | **-0.85** | **-0.57** | **-0.47** | **-0.43** | **0.14** | **-0.98** |
| Azs HELIX LRM3 | | **-1.33** | **-0.39** | **-1.09** | **0.23** | **1.34** | **-1.24** |

N: sample size; SD: standard deviation; Azs: adjusted z-scores; SA_Dent_: Dentine Surface Area; SA_Enam_: Enamel Surface Area; L_EDJ_: EDJ length; 2D AET: 2D Average Enamel Thickness; 2D RET: 2D Relative Enamel Thickness; BW: bicervical width.

→ **Both HELIX LM3s show values that are compatible with the healthy modern human variation.** Values falling outside the comparative range can be easily explained by the low resolution of the scan, which does not allow to accurately delineate the EDJ, and thus affect the EDJ length and the delimitation of the dentine core.

*C. 3D Enamel Thickness*

*C.1. 3D enamel thickness indices in the HELIX M3s*

For the 3D indices (3D AET and 3D RET), the segmentation of the dental tissues of the HELIX molar (See Fig. 1B and C) enables to calculate the enamel and dentine volumes, as well as the EDJ surface area. The 3D indices are calculated as follows:

3D AET = V_Enamel_ / SA_EDJ_

3D RET = 3D AET / (V_Dentine_^1/3) × 100

| Specimen | V_Dent_ [mm^3^] | V_Enam_ [mm^3^] | SA_EDJ_ [mm²] | 3D AET [mm] | 3D RET [scale free] |
| --- | --- | --- | --- | --- | --- |
| HELIX LRM3 | 144.86 | 224.05 | 130.96 | **1.71** | **14 .21** |

V_Cr_: whole Crown Surface Area; V_Dent_: Dentine Volume; V_Enam_: Enamel Volume; SA_EDJ_: EDJ surface area; 3D AET: 3D Average Enamel Thickness; 3D RET: 3D Relative Enamel Thickness.

*C.2. Comparison of the HELIX LRM3 to healthy modern M3s (source: Olejniczak et al., 2008) using adjusted z-scores ^15,16^.*

|  |  | V_Dent_ [mm^3^] | V_Enam_ [mm^3^] | SA_EDJ_ [mm²] | 3D AET [mm] | 3D RET [scale free] |
| --- | --- | --- | --- | --- | --- | --- |
| *Comparative sample of modern human M3*^18^ | | | | | | |
|  | N | 19 | 19 | 19 | 19 | 19 |
|  | Mean | 210.96 | 193.78 | 144.77 | **1.50** | **25.37** |
|  | SD | 43.19 | 65.76 | 41.31 | **0.24** | **7.77** |
| *Adjusted z-scores* | | | | | | |
| Azs HELIX LRM3 | | **-0.73** | **0.22** | **-0.16** | **0.40** | **-0.68** |

N: sample size; SD: standard deviation; Azs: adjusted z-scores; V_Dent_: Dentine Volume; V_Enam_: Enamel Volume; SA_EDJ_: EDJ surface area; 3D AET: 3D Average Enamel Thickness; 3D RET: 3D Relative Enamel Thickness.

→ **The HELIX LRM3 shows values falling within the healthy modern human variation.** As for the 2D indices, the volume of dentine appears relatively low because of the low resolution of the scan affecting the delimitation of the dentine core.

Overall, the HELIX LRM3 has normal distribution of enamel both in 2D and 3D, when compared to healthy modern human M3s.

**Cited references**

1. Martin L.B. 1985. Significance of enamel thickness in hominoid evolution. *Nature* **314**: 260–263.

2. Beynon A.D., M.C. Dean & D.J. Reid. 1991. On thick and thin enamel in hominoids. *American Journal of Physical Anthropology* **86**: 295–309.

3. Smith T.M., L.B. Martin & M.G. Leakey. 2003. Enamel thickness, microstructure and development in Afropithecus turkanensis. *Journal of Human Evolution* **44**: 283–306.

4. Beynon A. & B. Wood. 1986. Variations in enamel thickness and structure in East African hominids. *American Journal of Physical Anthropology* **70**: 177–193.

5. Buti L., A. Le Cabec, D. Panetta, *et al.* 2017. 3D enamel thickness in Neandertal and modern human permanent canines. *Journal of Human Evolution* **113**: 162–172.

6. Martin L.B., A.J. Olejniczak & M.C. Maas. 2003. Enamel thickness and microstructure in pitheciin primates, with comments on dietary adaptations of the middle Miocene hominoid Kenyapithecus. *Journal of Human Evolution* **45**: 351–367.

7. Feeney R.N.M., J.P. Zermeno, D.J. Reid, *et al.* 2010. Enamel thickness in Asian human canines and premolars. *Anthropol. Sci.* **118**: 191–198.

8. Gantt D.G. 1986. Enamel thickness and ultrastructure in hominoids: with reference to form, function and phylogeny. *Comparative Primate Biology* **1**: 453–475.

9. Grine F.E. & L.B. Martin. 1988. Enamel thickness and development in *Australopithecus* and *Paranthropus*. In *Evolutionary history of the “robust” australopithecines* Grine F.E., Ed. 3–42. New York, NY: Aldine de Gruyter.

10. Kono R.T. 2004. Molar enamel thickness and distribution patterns in extant great apes and humans: new insights based on a 3-dimensional whole crown perspective. *Anthropological Science* **112**: 121–146.

11. Shellis R.P., A.D. Beynon, D.J. Reid, *et al.* 1998. Variations in molar enamel thickness among primates. *Journal of Human Evolution* **35**: 507–522.

12. DeMiguel D., D.M. Alba & S. Moyà‐Solà. 2013. European pliopithecid diets revised in the light of dental microwear in Pliopithecus canmatensis and Barberapithecus huerzeleri. *American Journal of Physical Anthropology* **151**: 573–582.

13. Le Cabec A., T. Colard, D. Charabidze, *et al.* 2021. Insights into the palaeobiology of an early *Homo* infant: multidisciplinary investigation of the GAR IVE hemi-mandible, Melka Kunture, Ethiopia. *Scientific Reports* **11**: 23087.

14. Olejniczak A.J., T.M. Smith, M.M. Skinner, *et al.* 2008. Three-dimensional molar enamel distribution and thickness in *Australopithecus* and *Paranthropus*. *Biology Letters* **4**: 406–410.

15. Maureille B., H. Rougier, F. Houet, *et al.* 2001. Les dents inférieures du Néandertalien Regourdou 1 (site de Regourdou, commune de Montignac, Dordogne): Analyses métriques et comparatives. *PALEO. Revue d’archéologie préhistorique* **13**: 183–200.

16. Scolan H., F. Santos, A.-M. Tillier, *et al.* 2012. Des nouveaux vestiges néanderthaliens à Las Pélénos (Monsempron-Libos, Lot-et-Garonne, France). *Bulletins et mémoires de la Société d’anthropologie de Paris* **24**: 69–95.

17. Smith T.M., A.J. Olejniczak, D.J. Reid, *et al.* 2006. Modern human molar enamel thickness and enamel–dentine junction shape. *Archives of Oral Biology* **51**: 974–995.

18. Olejniczak A.J., P. Tafforeau, R.N.M. Feeney, *et al.* 2008. Three-dimensional primate molar enamel thickness. *Journal of Human Evolution* **54**: 187–195.
