# Supplementary figures and images for: Impact of claudin‐10 deficiency on amelogenesis: Lesson from a HELIX tooth
Source: Ann N Y Acad Sci. 2022 Jul 28;1516(1):197–211. doi: 10.1111/nyas.14865 (PMC9796262; doi:10.1111/nyas.14865)

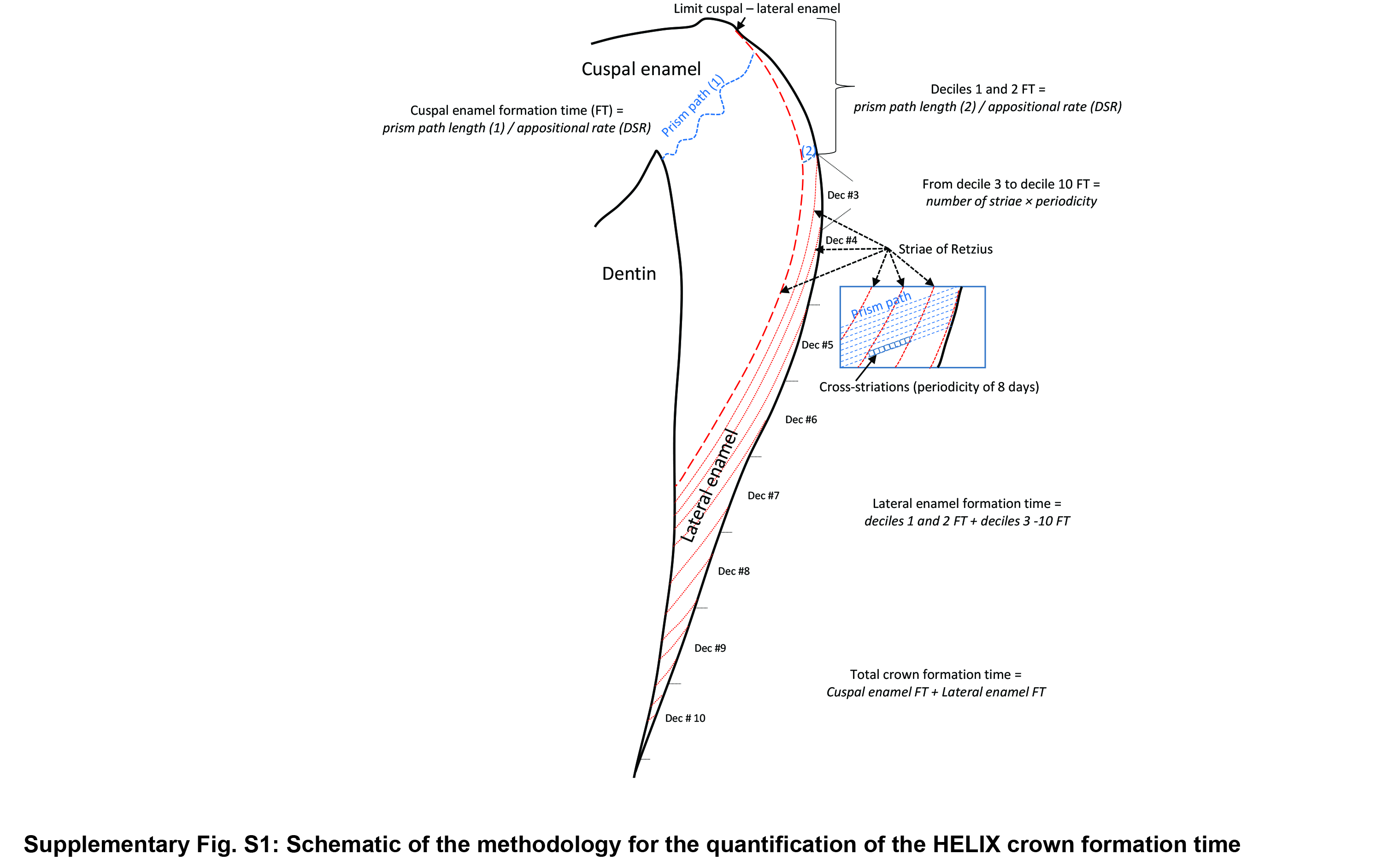

Supplement: Supplementary file 2 — Figure S1 Schematic of the methodology for the quantification of the HELIX crown formation time [file NYAS-1516-197-s009.tif]

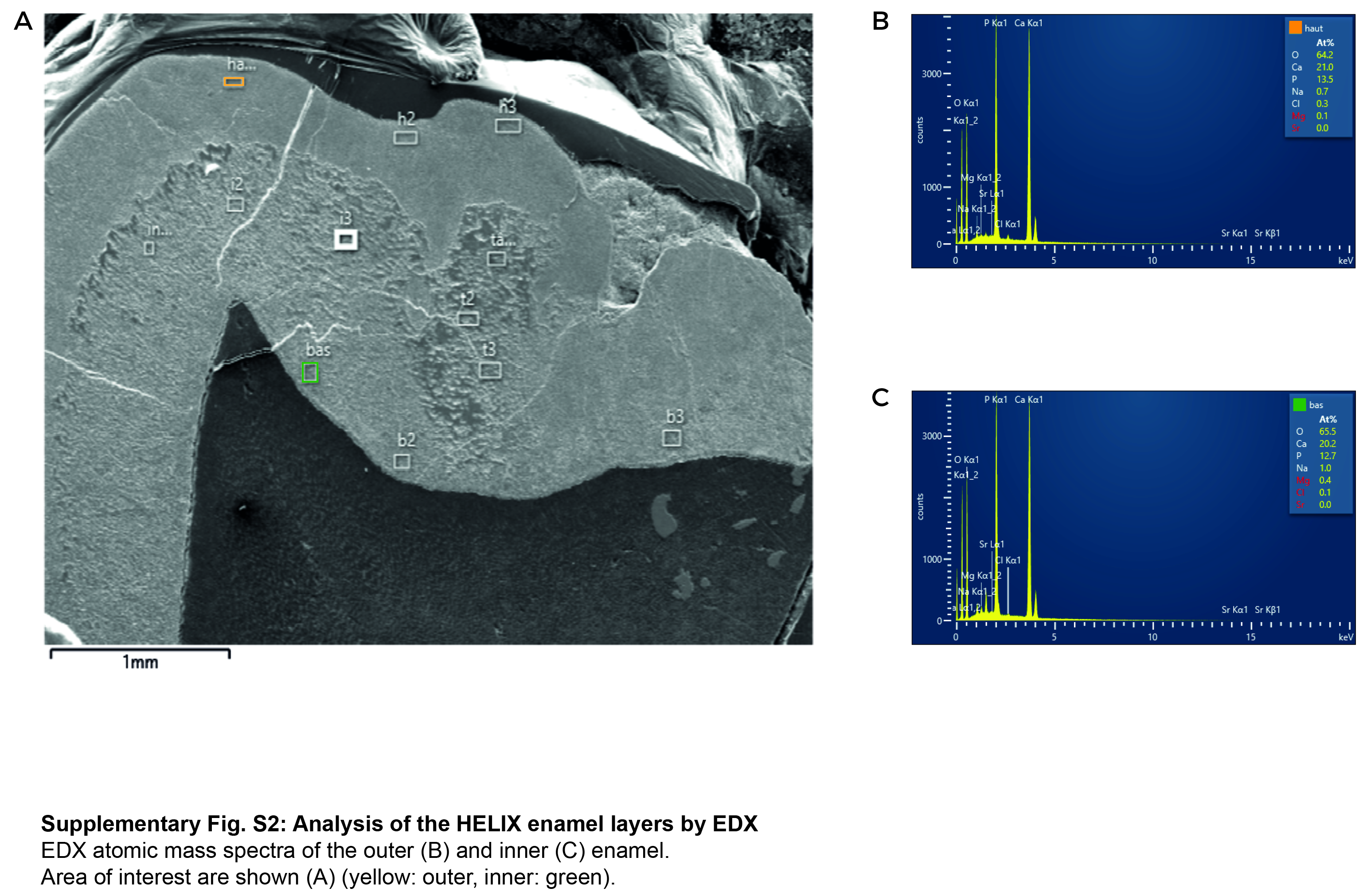

Supplement: Supplementary file 3 — Figure S2 Analysis of the HELIX enamel layers by EDX. EDX atomic mass spectra of the outer (B) and inner (C) enamel. Area of interest is shown (A) (yellow: outer, inner: green). [file NYAS-1516-197-s001.tif]

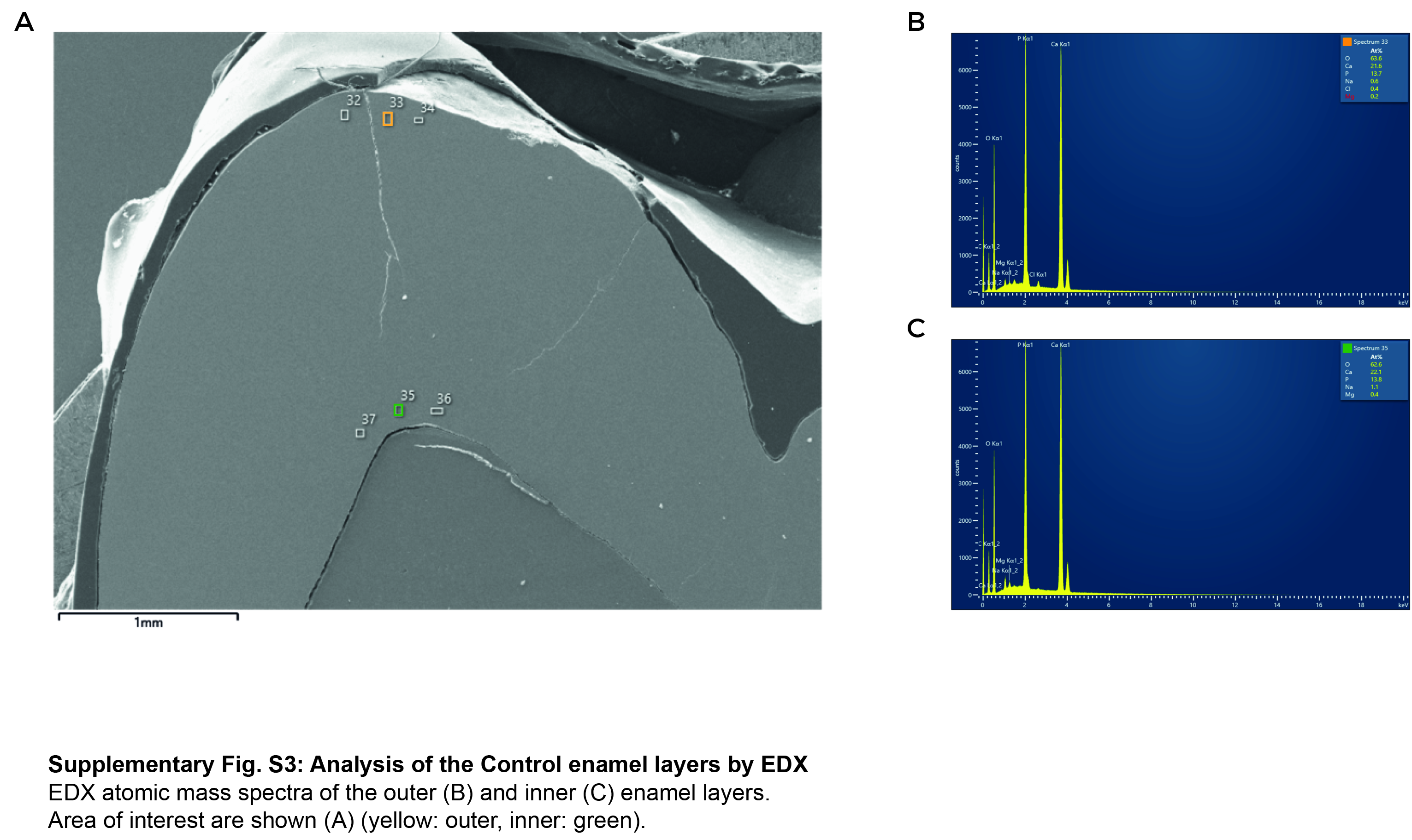

Supplement: Supplementary file 4 — Figure S3 Analysis of the control enamel layers by EDX. EDX atomic mass spectra of the outer (B) and inner (C) enamel layers. Area of interest is shown (A) (yellow: outer, inner: green). [file NYAS-1516-197-s006.tif]

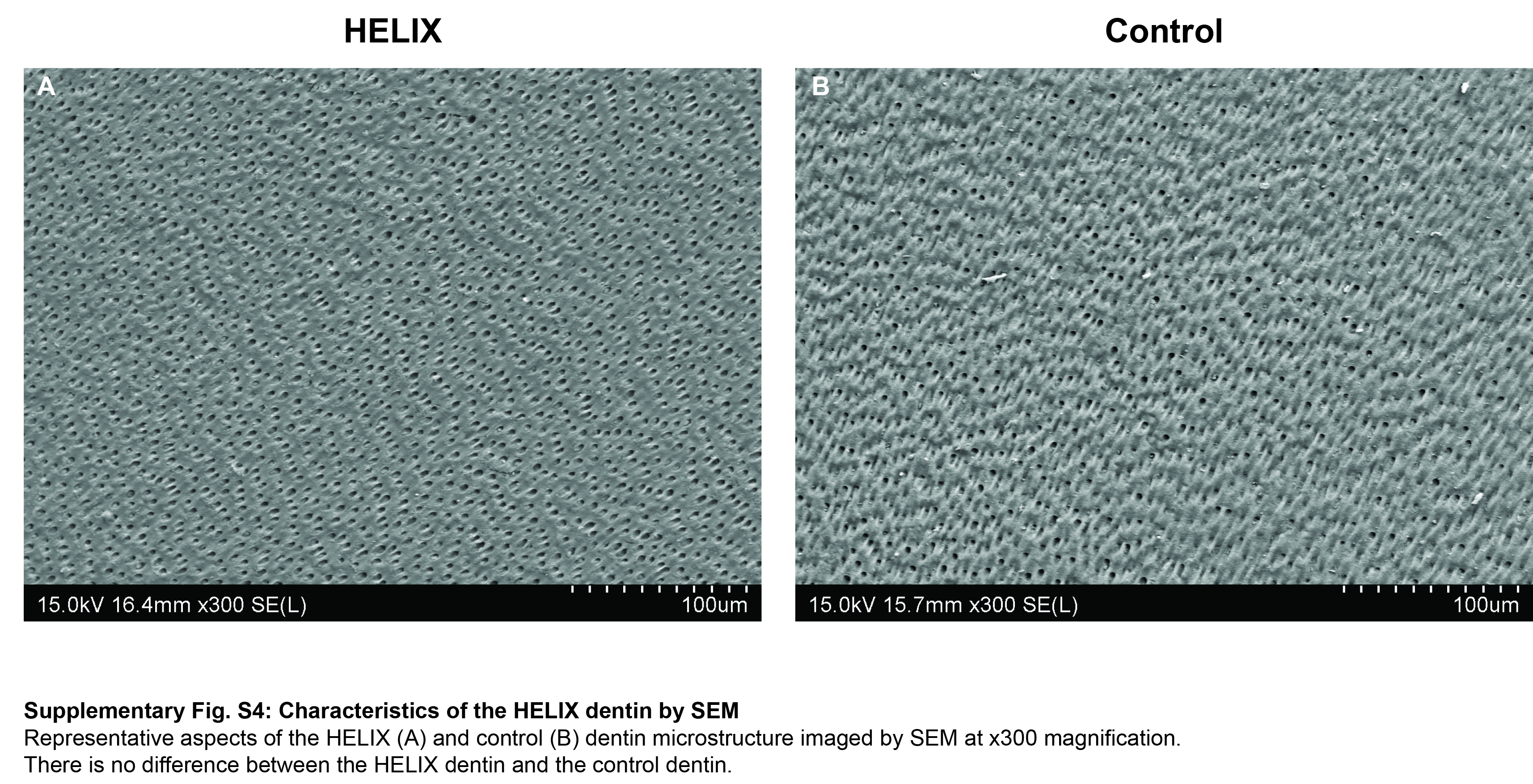

Supplement: Supplementary file 5 — Figure S4 Characteristics of the HELIX dentin by SEM. Representative aspects of the HELIX (A) and control (B) dentin microstructure imaged by SEM at x300 magnification. There is no difference between the HELIX dentin and the control dentin. [file NYAS-1516-197-s004.tif]

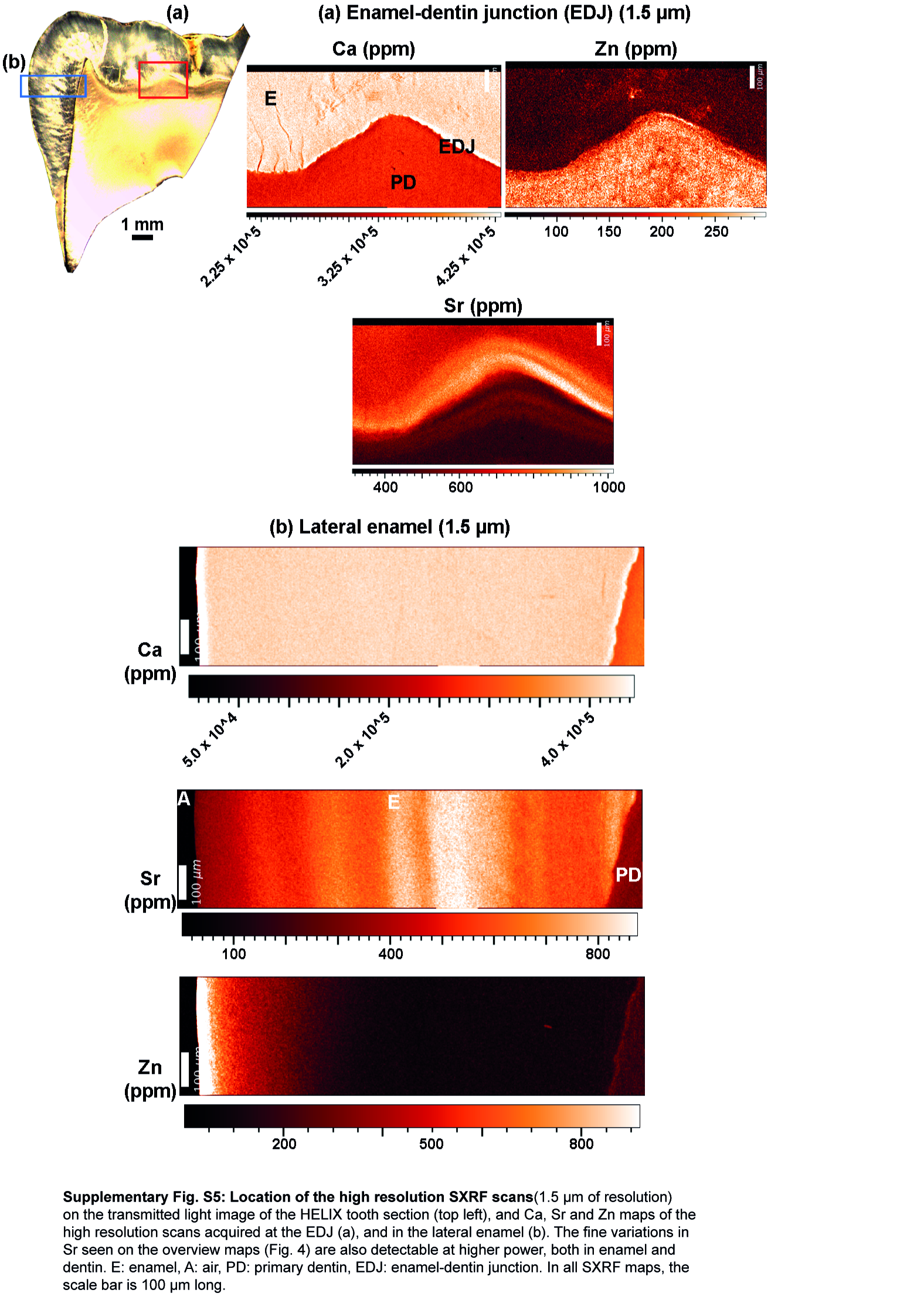

Supplement: Supplementary file 6 — Figure S5 Location of the high‐resolution SXRF scans (1.5 µm of resolution) on the transmitted light image of the HELIX tooth section (top left), and Ca, Sr, and Zn maps of the high‐resolution scans acquired at the EDJ (A), and in the lateral enamel (B). The fine variations in Sr seen on the overview maps (Figure 4) are also detectable at higher power, both in enamel and dentin. E, enamel; A, air; PD, primary dentin; EDJ, enamel–dentin junction. In all SXRF maps, the scale bar is 100 µm long. [file NYAS-1516-197-s007.tif]

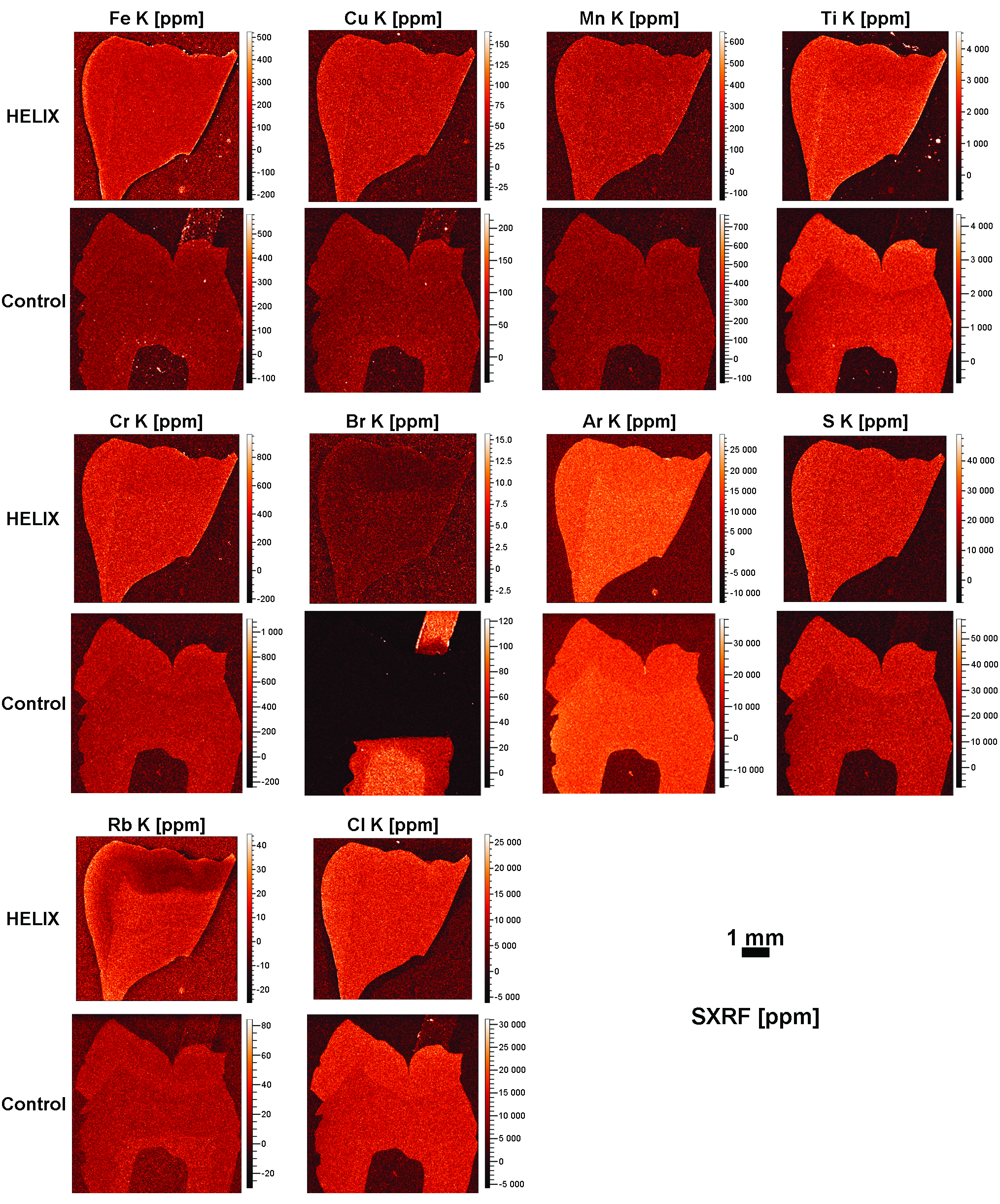

Supplement: Supplementary file 7 — Figure S6 SXRF characterization of the HELIX molar. SXRF characterization of the HELIX and control teeth for mapping Fe, Cu, Mn, Ti, Cr, Br, Ar, S, Rb, and Cl (SXRF overviews at 10 µm). In both the HELIX and control molar crowns, all these elements have a similar distribution with comparable ranges of concentration (in ppm). [file NYAS-1516-197-s002.tif]
