# Supplementary material for: Impact of claudin‐10 deficiency on amelogenesis: Lesson from a HELIX tooth
Source: Ann N Y Acad Sci. 2022 Jul 28;1516(1):197–211. doi: 10.1111/nyas.14865 (PMC9796262; doi:10.1111/nyas.14865)
Supplement: Supplementary file 8 — Table S1 Daily secretion rate (DSR) from the enamel–dentin junction (EDJ) to the outer enamel surface (OES) [file NYAS-1516-197-s003.docx]

**Table S1**: Daily secretion rate (DSR) from the enamel-dentin junction (EDJ) to the outer enamel surface (OES).

| **100-μm zone**  (distance in mm) | EDJ-0.1 | 0.1-0.2 | 0.2-0.3 | 0.3-0.4 | 0.4-0.5 | 0.5-0.6 | 0.6-0.7 | 0.7-0.8 | 0.8-0.9 | 0.9-1 | 1-1.1 | 1.1-1.2 | 1.2-1.3 | 1.3-OES |
| --- | --- | --- | --- | --- | --- | --- | --- | --- | --- | --- | --- | --- | --- | --- |
| **DSR** ( in µm/day) | 1.96 | 2.18 | 2.23 | 2.46 | 2.48 | 2.68 | 2.69 | 2.85 | 3.02 | 3.20 | 3.78 | 4.29 | 4.52 | 4.62 |
