# Supplementary material for: Impact of claudin‐10 deficiency on amelogenesis: Lesson from a HELIX tooth
Source: Ann N Y Acad Sci. 2022 Jul 28;1516(1):197–211. doi: 10.1111/nyas.14865 (PMC9796262; doi:10.1111/nyas.14865)
Supplement: Supplementary file 9 — Table S2 Cumulative formation time and distance from the outer enamel surface (OES) to the enamel–dentin junction (EDJ) of the major strontium (Sr) variations evidenced by synchrotron X‐ray fluorescence (SXRF) [file NYAS-1516-197-s005.docx]

| **Markers** | **EDJ** | **A** | **B** | **C** | **D** | **E** | **F** | **G** | **H (OES)** |
| --- | --- | --- | --- | --- | --- | --- | --- | --- | --- |
| **Cumulative distance (μm)** | 0 | 71 | 158 | 418 | 728 | 878 | 1033 | 1167 | 1336 |
| **Cumulative formation time of each Sr event (day)** | 0 | 37 | 103 | 224 | 372 | 431 | 480 | 530 | 564 |

**Table S2**: Cumulative formation time and distance from the outer enamel surface (OES) to the enamel dentin junction (EDJ) of the major Strontium (Sr) variations evidenced by Synchrotron X-Ray Fluorescence (SXRF).
